# Retraction Note: A novel positive feedback loop of linc02042 and c-Myc mediated by YBX1 promotes tumorigenesis and metastasis in esophageal squamous cell carcinoma

**DOI:** 10.1186/s12935-024-03397-z

**Published:** 2024-06-14

**Authors:** Jiahui Du, Guangzhao Zhang, Hongli Qiu, Haifeng Yu, Wuying Yuan

**Affiliations:** Department of Minimally invasive surgery, Henan Provincial Chest Hospital, No. 1 Weiwu Road, Jinshui District, Zhengzhou, 450000 People’s Republic of China

**Retraction Note: Cancer Cell International (2020) 20:75**

10.1186/s12935-020-1154-x

The Editor-in-Chief has retracted this article after concerns were raised about the data reported. Specifically:

- In Fig. 2F, the panels for sh-linc02042#1 and sh-linc02042#1 both appear highly similar to panels previously published in a paper with no common authors [1], where they are described differently.
- The blot for KYSE30 in Fig. 4B and the β-actin blot for KYSE30 in Fig. 4D each appear highly similar to blots previously published in a paper with no common authors [2], where they are described differently.

The Editor-in-Chief no longer has confidence in the reliability of the article’s results and conclusions.

The authors did not respond to correspondence from the publisher about this retraction.
